# Health Care Resources and 24,910 Deaths Due to Traffic Accidents: An Ecological Mortality Study in Poland

**DOI:** 10.3390/ijerph18115561

**Published:** 2021-05-22

**Authors:** Agnieszka Genowska, Jacek Jamiołkowski, Krystyna Szafraniec, Justyna Fryc, Andrzej Pająk

**Affiliations:** 1 Department of Public Health, Medical University of Bialystok, 15-295 Bialystok, Poland; 2 Department of Population Medicine and Lifestyle Diseases Prevention, Medical University of Bialystok, 15-269 Bialystok, Poland; jacek.jamiolkowski@umb.edu.pl; 3 Department of Epidemiology and Population Studies, Jagiellonian University Medical College, 31-066 Krakow, Poland; krystyna.szafraniec@uj.edu.pl (K.S.); andrzej.pajak@uj.edu.pl (A.P.); 4 Faculty of Medicine, Medical University of Bialystok, 15-540 Bialystok, Poland

**Keywords:** traffic accidents, health inequalities, out-of-hospital deaths, hospitalized fatal cases, health care disparities

## Abstract

Background: Deaths due to traffic accidents are preventable and the access to health care is an important determinant of traffic accident case fatality. This study aimed to assess the relation between mortality due to traffic accidents and health care resources (HCR), at the population level, in 66 sub-regions of Poland. Methods: An area-based HCR index was delivered from the rates of physicians, nurses, and hospital beds. Associations between mortality from traffic accidents and the HCR index were tested using multivariate Poisson regression models. Results: In the sub-regions studied, the average mortality from traffic accidents was 11.7 in 2010 and 9.3/100.000 in 2015. After adjusting for sex, age and over time trends in mortality, out-of-hospital deaths were more frequently compared to hospitalized fatal cases (incidence rate ratio (IRR) = 1.68, 95% CI 1.45–1.93). Compared to sub-regions with high HCR, mortality from traffic accidents was higher in sub-regions with low and moderate HCR (IRR = 1.25, 95% CI 1.11–1.42 and IRR = 1.19, 95% CI 1.02–1.38, respectively), which reflected the differences in out-of-hospital mortality most pronounced in car accidents. Conclusions: Poor HCR is an important factor that explains the territorial differentiation of mortality due to traffic accidents in Poland. The high percentage of out-of-hospital deaths indicates the importance of preventive measures and the need for improvement in access to health care to reduce mortality due to traffic accidents.

## 1. Introduction

Injuries from traffic accidents due to the dangerous behaviors of pedestrians and drivers are one of the main causes of death in most countries. Traffic accident-related mortality is unevenly distributed geographically, with Western European countries having much lower rates than Central and Eastern European countries [1,2,3]. Fatality rate due to traffic accidents in Poland is almost twice as high as the average in the European Union, and three times as high as Sweden, the United Kingdom, and the Netherlands, who are leaders in road safety [4]. Traffic accidents cause significant economic losses to the whole society, as a consequence of medical and administrative costs incurred, as well as the productivity loss among the injured and deceased. In Poland, the cost of a road accident corresponding to one fatality has been estimated at 561 thousand euros and the total social cost at €10.5 billion (i.e., 2.1% of gross domestic products). These high costs primarily result from the loss of productivity associated with premature mortality due to accidents in the 25–59 age group, which account for about 50% of the total number of accident-related deaths [5].

In addition to poor safety standards and drunkenness influencing the behavior of road users, a significant factor that increases the risk of a fatal traffic accident is the incompliance of vehicle speeds with road traffic rules [6,7,8]. High speeds cause injuries, mainly severe head and chest injuries from contact with vehicle components or road infrastructure. Recent studies from Germany [9] and the USA [10] have highlighted the relationship between an accident site in rural areas and the risk of death. Due to their geographical location, less urbanized areas may be characterized by longer waiting times for medical assistance or limited access to adequate out-of-hospital care or transportation to the hospital, which affects survival [11]. Some studies also indicate that the risk of death due to traffic accidents may be influenced by factors such as age and male gender, and that pedestrians are also a high-risk group [12,13].

Although there has been a significant reduction in traffic accident mortality in the last three decades, due to improvements in road systems, education, and prevention campaigns [14], the causes of relatively high mortality in Central and Eastern European countries have not been clearly explained thus far. Only a few studies have compared the mortality rate in Poland with other European countries due to the lack of obligation to collect relevant data on mortality associated with road accident injuries [15]. The existing studies are just descriptive [16,17]. However, identifying the factors related to road accident mortality may be of interest in countries such as Poland, which are characterized by insufficient medical care leading to unintentional injury deaths that can be avoided [18]. Poor access to health care resources has tragic consequences and increases the risk of mortality due to traffic accidents [19]. There is little information about which areas have poor access to health care in Poland, and to what extent these poor resources are affected by the adverse effects of accidents. It would be important to investigate whether sub-regions with poor access to health care will have more out-of-hospital deaths, and therefore higher mortality from traffic accidents [9,11,20].

The purpose of the study was to assess the relation between mortality due to traffic accidents and health care resources (HCR), at the population level, in 66 sub-regions of Poland.

## 2. Materials and Methods

### 2.1. Study Design

This is an ecological-type study, in which annual data on HCR and deaths due to transport accidents collected from 66 administrative areas of Poland were analyzed. These areas were defined as sub-regions in the Nomenclature des Unités Territoriales Statistiques (NUTS-3) in 2006 [21]. In general, the number of residents in sub-regions ranges from 277,241 to 1,721,386 (average 583,366). The information analyzed for the years 2010–2015 was obtained from the Central Statistical Office.

### 2.2. Mortality

The study analyzed the data on deaths due to transport accidents. The individual categories of transport accidents have been coded by the International Statistical Classification of Diseases and Related Health Problems, Tenth Revision (ICD-10), as transport accidents in total (codes V01–V99), including accidents with participation of pedestrian (V01–V09), pedal cyclist (V10–V19), motorcycle rider and three-wheeled motor vehicle (V20–V39), car (V40–V49), and heavy transport vehicle (V50–V97), and other and unspecified transport accidents (V98–V99) [22].

The study analyzed the age-standardized mortality due to transport accidents. A direct standardization method that considers the demographic structure in 5-year age groups was used for separately analyzing the data for each of the 66 sub-regions of Poland. As a standard population, we assumed the population of Poland in 2010, for which standardized mortality rates were determined [23]. In addition to these rates, 95% confidence intervals (CIs) were calculated.

Based on the place of death, deaths due to traffic accidents were classified into (1) out-of-hospital deaths; and (2) fatal hospitalized cases, which included those that occurred in a hospital or long-term care institutions [11].

Changes in mortality rates during the period 2010–2015 were described with the deaths prevented or postponed (DPP) index [24]. The value of this index was calculated as the percentage of the difference between the expected number of deaths in 2015 (assuming the same mortality rate as in 2010) and the actual number of deaths in 2015 relative to the expected number of deaths in 2015:

(1) $$DPP=\frac{deaths\;expected_{2015}-deaths\;observed_{2015}}{deaths\;expected_{2015}\;}\cdot 100\%$$

### 2.3. Area-Based Health Care

Health care was assessed in each sub-region using the HCR index, which was created on the basis of all human and infrastructural resources potentially affecting access to health care. Three factors were included in this index: number of physicians per 10,000 population, number of nurses per 10,000 population, and number of hospital beds per 10,000 population. To calculate the HCR index, its components—variables—were normalized by a linear transformation in such a way that their expected value was equal to 0 and standard deviation was equal to 1. Finally, the arithmetic mean of the transformed components was taken as the index value [25]. The HCR index was calculated based on the average values of the component variables for the years 2010–2015. The physicians and nurses ratio for 2012 was calculated from the average values of 2011 and 2013 due to the lack of data. Additionally, the 66 sub-regions were divided into three groups based on the tercile distribution of HCR index: the group with the lowest index and thus the lowest level of HCR (low), the group with the highest index (high), and the intermediate group (moderate).

### 2.4. Statistical Analysis

To determine the relationship between the traffic accident-related mortality and the HCR index, we used the Poisson regression model for count data. Calculations were realized using generalized linear models, with logarithm as a link function and Poisson as a probability distribution, including the number of people in sub-regions as the offset.

Due to repeated measurements in the same statistical units (traffic accident mortality in sub-regions in subsequent years during the period 2010–2015), the generalized estimating equations (GEE) framework was used for obtaining generalized linear models for correlated data [26]. An exchangeable structure was assumed for the working correlation matrix in the GEE models.

The Poisson models were presented using the regression coefficients that were exponentially transformed, so they can be interpreted as the expected relative change of the dependent variable (i.e., the number of deaths due to traffic incidents) calculated for an increase of the independent variable by 1 unit (i.e., incidence rate ratio (IRR) = e^β^). Two types of multivariate models were evaluated. The first model included the index of HCR at the sub-region level, variables describing demographic category (gender, age), and the year observation as independent variables. Depending on the place of death, different categories of deaths were analyzed in separate models as follows: out-of-hospital deaths, hospitalized fatal cases, and in total. In the second model, all traffic accident deaths were included with the index of HCR as the independent variable and an additional variable describing the place of death. This model was adjusted for gender, age, and the observation year. The results are presented as IRR with 95% CIs and p-values of corresponding Wald’s test.

Statistical calculations were performed using IBM^®^ SPSS^®^ Statistics for Windows, version 20.0 statistical package (IBM Corporation, Armonk, NY, USA). The significance level was assumed as α = 0.05 in statistical tests.

## 3. Results

In the period 2010–2015, a total of 24,910 deaths due to transport accidents were recorded in Poland, which included 19,367 deaths among men (77.7%). Accidents in the age groups 25–44 (29.6%) and 45–64 (31.3%) were found to be dominant. Accidents involving cars (36.4%) and pedestrian (31.1%) were the most frequent. A high percentage of deaths due to transport accidents occurred out of hospital (65.2%) and the remaining occurred in hospital (34.1%) or long-term care institutions (0.7%).

**Figure 1:**
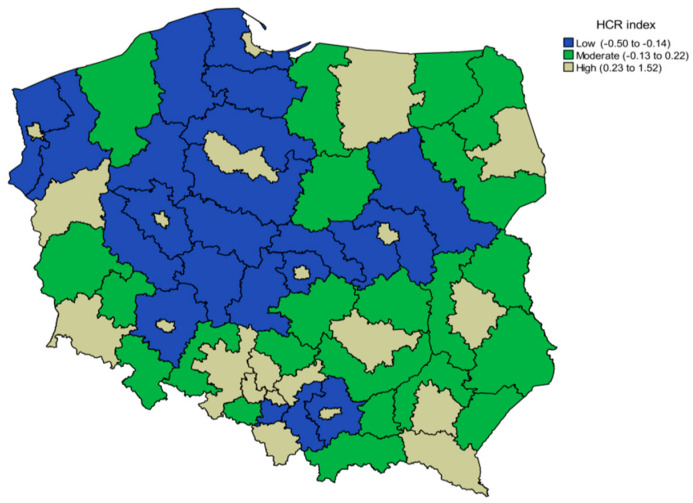
Geographic variation of the mean of health care resources (HCR) index for the years 2010–2015 in 66 sub-regions of Poland.

In the sub-regions studied, the range for low, moderate and high HCR index, assigned by the terciles for the whole sample, were: −0.50 to −0.14, −0.13 to 0.22 and 0.23 to 1.52, respectively (according to the average for years 2010–2015). The territorial distribution of sub-regions by HCR is presented in Figure 1. Sub-regions with the highest HCR index were primarily characterized by large urban agglomerations. A substantial difference in population density was noted, which was on average 124 people/km^2^ in the sub-regions with low HCR while it was over sevenfold higher (923 people/km^2^) in the sub-regions with high HCR, which indicates that high HCR index are mainly linked to highly urbanized areas.

In the group of sub-regions with high HCR, there were about two times more hospital beds and physicians compared to sub-regions with low HCR, but only 30% more nurses. The highest mortality rate due to traffic accidents (in total) was noted in the sub-regions with low HCR (11.36/100,000) and moderate HCR (11.60/100,000), and the deaths were mostly out-of-hospital ones (8.65/100,000). Among the analyzed accident categories, the highest burden was caused by those involving cars, and high mortality rates occurred in the sub-regions with low and moderate HCR (in total: 4.85/100,000 and 4.46/100,000, respectively). In sub-regions with high HCR, accidents involving pedestrians were dominant (in total: 2.92/100,000), whereas the lowest mortality rates were related to accidents involving pedal cyclists. Generally, mortality in sub-regions with high HCR was lower compared to sub-regions with low HCR, with the exception of other and unspecified transport accidents, which were the lowest in these sub-regions (in total: 0.96/100,000) (Table 1).

**Figure 2:**
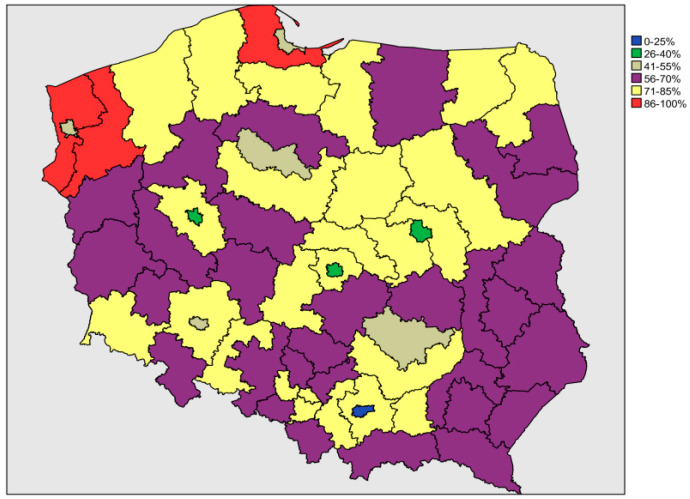
Geographic variation of the mean percentage of out-of-hospital deaths due to transport accidents for the years 2010–2015 in 66 sub-regions of Poland.

The mean percentage of out-of-hospital deaths due to transport accidents for the period 2010–2015 ranged from 23.7% in the Kraków sub-region to 96.3% in the Szczeciński sub-region (Figure 2).

The total standardized mortality rate due to transport accidents was the highest in the sub-regions with the lowest values for the HCR index, whereas it was lowest in the sub-regions with the highest values of the index. The age standardized rate of out-of-hospital deaths was the highest in the sub-regions with low HCR (9.25/100,000 in 2010 and 7.81/100,000 in 2015). The value of both these rates was about threefold higher than hospitalized fatal cases in the sub-regions with low HCR. Likewise, in the sub-regions with moderate HCR, the disproportion of deaths was twofold, while in the sub-regions with high HCR the out-of-hospital deaths and hospitalized fatal cases were at a similar level. A comparison of hospitalized fatal cases between the groups of sub-regions showed that the number of these cases was 1.5-fold higher in the sub-regions with high HCR than those with low HCR (Table 2).

No significant linear trend was observed in the percentage of prevented or postponed deaths related to transport accidents between all three sub-region groups defined by HCR index (*p* = 0.080). Similarly, no significant differences were found for out-of-hospital deaths (*p* = 0.140) and hospitalized fatal cases (*p* = 0.182). Between years 2010 and 2015, the total mortality due to transport accidents decreased by 21.4%, but the changes in hospitalized fatal cases were higher (27.7%) in comparison to out-of-hospital deaths (17.5%) (Table 2).

A significant relationship was found between the HCR index and total mortality due to transport accidents (Table 3). In the sub-regions with low HCR, there was 25% more traffic accident-related mortality compared to the sub-regions with high HCR (IRR = 1.25, 95% CI 1.09, 1.43, *p* ≤ 0.01). The effect was slightly lower for the sub-regions with moderate HCR compared to those with high HCR (IRR = 1.21, 95% CI 1.03, 1.42, *p* ≤ 0.05). Similarly significant relationships were found in cases involving pedal cyclists and cars, and partially in cases involving pedestrians (only low HCR vs. high HCR) and heavy transport vehicles (only moderate HCR vs. high HCR). The accident mortality was higher among men than women (IRR = 3.64, 95% CI 3.49, 3.80, *p* ≤ 0.001), and a significant gender relationship was found to persist in each accident category. Age was also associated with mortality in traffic accidents, with the highest increase in deaths (516%) noted in the oldest age group (i.e., over 64 years of age) compared to the youngest age group (i.e., under 15 years of age) (IRR = 5.16, 95% CI 4.60, 5.80, *p* ≤ 0.001). Similar relationships for pedestrians and pedal cyclists were observed in the older age group. In case of accidents involving motorcycle riders and three-wheeled motor vehicles, cars, and heavy transport vehicles, and other and unspecified transport accidents, compared to those in the 0–14 age group, the highest proportion of mortality occurred among young adults aged 15–24 years.

The pattern of the increased proportion of total mortality due to traffic accidents was reflected in the out-of-hospital deaths, especially in the category of accidents involving cars. On the other hand, for hospitalized fatal cases, a significant reduction of total mortality due to traffic accidents by 25% related to HCR occurred in sub-regions with low HCR compared to those with high HCR (IRR = 0.75, 95% CI 0.65, 0.87, *p* ≤ 0.001), by 14% in sub-regions with moderate HCR compared with high HCR (IRR = 0.86, 95% CI 0.75, 0.99, *p* ≤ 0.05), as well as among accidents involving pedestrians (by 20%), and other and unspecified transport accidents (by 41%). The mortality related to HCR among pedal cyclists was higher by 25% (in sub-regions with low HCR vs. those with high HCR) and 34% (in sub-regions with moderate HCR vs. those with high HCR), as well as for accidents involving heavy transport vehicles (by 33% in sub-regions with moderate HCR vs. those with high HCR).

A statistically significant decrease in mortality from transport accidents by 5.0% annually was found for both out-of-hospital deaths and hospitalized fatal cases combined. Among the accident categories, a significant decrease was noted in cases involving pedestrians and cars in total mortality (by 5.0%/year and by 7.0%/year, respectively), such as in out-of-hospital deaths (by 5.0%/year and by 6.0%/year, respectively) and hospitalized fatal cases (by 4.0%/year and by 5.0%/year, respectively). Reduced mortality trends were noted for traffic accidents involving motorcycle rider and three-wheeled motor vehicle as well as heavy transport vehicles (in total mortality: by 3.0%/year and by 7.0%/year, respectively; and hospitalized fatal cases: by 3.0%/year and by 5.0%/year, respectively). No significant changes were observed in mortality from accidents involving pedal cyclist and other and unspecified transport accidents (Table 3).

Table 4 presents results of the model including the place of death and HCR adjusted for sex, age, and mortality over time. The results indicate that the place of death affects mortality due to accidents more than HCR, except for the categories of accidents involving pedal cyclist and other and unspecified transport accidents. Out-of-hospital deaths due to traffic accidents were in total 68% higher compared to hospitalized fatal cases in total (IRR = 1.68, 95% CI 1.45, 1.93, *p* ≤ 0.001). The mortality in sub-regions with low HCR was 25% higher compared to those with high HCR (IRR = 1.25, 95% CI 1.11, 1.42, *p* ≤ 0.001) and 19% higher in sub-regions with moderate HCR compared to those with high HCR (IRR = 1.19, 95% CI 1.02, 1.38, *p* ≤ 0.05). Similar relationships were found for accidents involving pedestrians, motorcycle riders and three-wheeled motor vehicles, and cars. Among the cases involving pedal cyclists, the opposite relationship was found: mortality was 28% higher in sub-regions with moderate HCR compared to those with high HCR and 24% higher in sub-regions with low HCR compared to those with high HCR. Moreover, among cases involving pedal cyclists, the increase in out-of-hospital deaths of 10% was lower compared to the other accident categories. In the case of heavy transport vehicles, there was dependence only with the out-of-hospital deaths and the level of HCR was negligible. For other and unspecified transport accidents, however, a 32% reduction in mortality (IRR = 0.68, 95% CI 0.50, 0.94, *p* ≤ 0.05) was found in sub-regions with low HCR compared to those with high HCR.

## 4. Discussion

### 4.1. Main Findings

In the 66 sub-regions of Poland, large geographical variation was observed in mortality due to traffic accidents and in HCR. A low HCR was associated with an increased mortality due to traffic accidents, especially in the case of out-of-hospital mortality. There were more fatal hospitalized cases in the areas with high HCR. The relationship between traffic accident mortality and HCR was significant after controlling for gender, age, and changes in mortality over time. For accidents involving motorcycle riders, three-wheeled motor vehicles, cars, and heavy transport vehicles and other or unspecified transport accidents, the highest out-of-hospital mortality was found in adolescents. For accidents involving pedestrians and pedal cyclists, the highest mortality was observed in the elderly.

### 4.2. Interpretation of Results

The results obtained are similar to those reported by studies conducted in other countries, despite the use of different methods for estimation [9,11,12,13,20]. The inverse association between HCR and mortality due to traffic accidents in the out-of-hospital setting can be explained by the unequal distribution of HCR, which results in differences in access to medical intensive care units. The high percentage of out-of-hospital deaths observed in our study seems to be related to weak access to medical care.

The higher out-of-hospital mortality rate, especially in the case of car accidents, shows that the severity of these accidents is determined by the specific infrastructure conditions of the accident site. These accidents often occur in less urbanized areas where drivers develop high speeds. The results of studies by other authors [9] indicate that as much as 72% of deaths on-scene occur in the countryside. In general, victims of traffic accidents from rural areas have worse survival rates due to extended emergency response times and/or excessive waiting times [10,11]. Available studies indicate that a distance of more than 30 km from the trauma care center increases the risk of deaths due to accidents [27]. Patients with serious injuries are more likely to die on-scene before receiving peri-resuscitation care [20]. Air transport, which almost triples the survival rate compared to ambulance transport, is of great importance in caring for accident victims from rural areas [19,28].

In model 2, the increase in the rate of out-of-hospital deaths due to traffic accidents was lower compared to the results obtained from the care index for the category of pedal cyclist accidents, although in the other accident categories the increase in deaths was higher in relation to low and moderate HCR. It is likely that in the case of cyclist deaths, other features may play a greater role in increasing mortality when the level of HCR is low or moderate. However, it should be noted that areas with low levels of HCR are also less populated and have an imperfect infrastructure of bicycle paths. The higher mortality of cyclists in the areas with low- and moderate HCR may be related to these factors, which suggests that the HCR index may also be a marker of other factors connected with mortality due to traffic accidents [29].

Our results showed gender-related health inequalities, with significantly higher mortality rates in men, as confirmed by other studies [13,30]. Risky driving behaviors among men are common at an early age and are observed throughout the life cycle. Men are more likely to drive after drinking alcohol, use mobile phones while driving, and not to use safety devices (helmets, seatbelts), which can lead to fatal injuries. In this study, another factor clearly related to higher mortality due to traffic accidents was older age, which is mainly associated with visual and cognitive impairment. Moreover, in older people, osteoporosis and reduced organ function contribute to more severe injuries during accidents, especially when the victims are pedestrians and cyclists [12,31]. High mortality was also observed among young adults (15–24 years old) in traffic accidents involving motorcycle rider and three-wheeled motor vehicle, car, and heavy transport vehicle and other and unspecified transport accidents. This risk is often related to driving under the influence of alcohol or drugs, which, when combined with the lack of driving experience, results in tragic consequences [32,33].

Patterns of mortality due to traffic accidents differed territorially, with the highest mortality rates recorded in sub-regions with low levels of HCR, which were located in the northern part of Poland. In these areas, more out-of-hospital deaths occurred, due to health care disparities. In the sub-regions of high HCR (large urban agglomerations), out-of-hospital mortality was lower compared to the low HCR sub-regions. This was due not only to better access to care, but also to the possibility of implementing preventative measures, more frequent traffic safety checks, and better road infrastructure, including traffic flow separators, better street lighting, and stepped bends [20,34,35,36]. Nevertheless, in urbanized areas, the so-called late deaths are more frequent than in rural areas.

The results of this study showed a significant reduction in mortality due to traffic accidents between 2010 and 2015. These positive changes may have resulted from improvements in the provision of medical equipment and apparatus to emergency services and from shorter waiting times for assistance due to the spread of mobile telephony. In addition, actions concerning the enforcement of traffic regulations and imposition of fines as well as the implementation of regulations on the technical conditions of vehicles played an important role [8]. However, no changes were seen in the mortality rate in the cases of accidents involving pedal cyclists, which may be related to the increased use of bicycles and inadequate infrastructure of bicycle paths, including improper marking and poor maintenance [37]. Evidence shows that in Poland a high fatality rate due to accidents involving pedal cyclists is associated with the use of roads during darkness [38]. This type of death can be avoided through national or local activities such as public education, investment in road infrastructure, law enforcement, and medical intensive care units [39].

### 4.3. Strengths and Limitations

According to our knowledge, this study is the first to assess the relationship between mortality due to traffic accidents and HCR in Poland using the area-level index. The information provided by the area-based HCR index may prove useful in identifying areas in need of health interventions. In addition, only a few studies have been conducted assessing avoidable deaths (DPP), most of which take place out-of-hospital [40]. Our analysis included a unique set of data on deaths related to all traffic accidents in sub-regions defined by the NUTS-3 classification used in the European Union [21].

The following limitations should be taken into account in interpreting the results of this study. First of all, this ecological study was carried out at the population level, and cannot determine the cause, mechanism and manner of death. Secondly, the data for analysis were collected from only one source (Central Statistical Office) and the study did not include information on the type of injuries or medical interventions performed. As research by other authors showed, the duration of the pre-hospital phase varies by the type of injury, which particularly applies to victims of penetrating injuries [41,42,43]. Besides this, in our study the assessment of HCR was based on general information by physicians, nurses, and hospitals only. More detailed information was not available in the routine statistics available for sub-regions in Poland, which in consequence could result in an increased influence of variation on the study results. Further, deaths due to accidents are assigned to the sub-region according to the place of death. In some sub-regions (mainly in the neighborhood of the large towns) large proportions of traffic accident victims are transported for hospitalization out of their area. This might increase proportion of out-of-hospital deaths in such sub-regions. Finally, classification into the HCR classes was done according to the six year mean of the HCR index. The index could have changed slightly overtime but without important impact for classification into the “low”, “medium” and “high” categories.

In addition, we had no information on how many accident victims received delayed medical assistance on the scene eventually died in inpatient care due to hemodynamic instability. This suggests that the results obtained may be underestimated. Moreover, the relationship between the out-of-hospital mortality and total accident mortality may be influenced by unknown factors, such as road conditions and speed of travel, weather, use of seat belts and helmets, and the impact of alcohol, which determine the severity of accidents [6,7,8]. Nevertheless, the analysis shows that the level of HCR is related to mortality due to traffic accidents.

## 5. Conclusions

Poor HCR is an important factor that explains the territorial differentiation of mortality due to traffic accidents in Poland. The high percentage of out-of-hospital deaths indicates the importance of preventive measures and the need for improvement in access to emergency medical services and intensive care units to reduce mortality due to traffic accidents.

## Figures and Tables

**Table 1:** Descriptive statistics for the health care and for deaths due to type of traffic accidents by health care resources (HCR) index in 66 sub-regions of Poland averaged over the period 2010–2015.

Index of Health Care Resources (Terciles)	Low HCR = −0.50 to −0.14	Moderate HCR = −0.13 to 0.22	High HCR = 0.23–1.52
Index components [n/10,000 population]			
Physicians	13.07	16.60	33.50
Nurses	33.60	46.56	43.13
Hospital beds	33.74	43.13	64.62
Out-hospital deaths due to traffic accidents [n/100,000 population]			
Pedestrian	2.63	2.23	1.49
Pedal cyclist	0.51	0.44	0.20
Motorcycle rider and three-wheeled motor vehicle	0.51	0.45	0.34
Car	3.81	3.29	1.77
Heavy transport vehicle	0.60	0.42	0.41
Other and unspecified transport accidents	0.58	1.02	1.02
All accidents	8.65	7.85	5.24
Hospitalized fatal cases of traffic accidents [n/100,000 population]			
Pedestrian	0.80	1.19	1.42
Pedal cyclist	0.25	0.35	0.27
Motorcycle rider and three-wheeled motor vehicle	0.16	0.22	0.28
Car	1.03	1.18	1.05
Heavy transport vehicle	0.09	0.11	0.20
Other and unspecified transport accidents	0.38	0.69	1.10
All accidents	2.72	3.75	4.33
Deaths due to traffic accidents in total [n/100,000 population]			
Pedestrian	3.43	3.43	2.92
Pedal cyclist	0.77	0.79	0.48
Motorcycle rider and three-wheeled motor vehicle	0.67	0.67	0.62
Car	4.85	4.46	2.82
Heavy transport vehicle	0.69	0.54	0.62
Other and unspecified transport accidents	0.96	1.71	2.12
All accidents	11.36	11.60	9.57

**Table 2:** Age-standardized mortality rates and deaths prevented or postponed due to transport accidents in Poland in the years 2010 and 2015 sub-region classes according to health care resources (HCR).

Index of Health Care Resources (Tercile)	Low		Moderate		High		Total	
Calendar year	2010	2015	2010	2015	2010	2015	2010	2015
Population number	12,256,046	12,407,445	11,567,978	11,414,694	14,705,842	14,615,100	38,529,866	38,437,239
Out-of-hospital deaths due to traffic accidents								
SMR (95% CI)	9.25 (8.71–9.79)	7.81 (7.31–8.32)	7.89 (7.38–8.40)	6.44 (5.97–6.91)	5.42 (5.05–5.80)	4.47 (4.12–4.82)	7.37 (7.09–7.64)	6.13 (5.88–6.38)
Observed number of deaths	1124	942	909	730	805	639	2838	2311
Expected number of deaths *		1121		898		783		2802
Number (percentage) of DPP relative to expected deaths		179 (16.0%)		168 (18.7%)		144 (18.4%)		491 (17.5%)
Linear trend for DPP	*p* = 0.140							
Hospitalized fatal cases of traffic accidents								
SMR (95% CI)	3.49 (3.15–3.82)	2.26 (1.99–2.53)	4.12 (3.75–4.50)	3.23 (2.90–3.56)	5.29 (4.92–5.66)	3.78 (3.46–4.10)	4.36 (4.15–4.57)	3.12 (2.94–3.30)
Observed number of deaths	414	275	476	375	790	564	1680	1214
Expected number of deaths *		416		475		788		1679
Number (percentage) of DPP relative to expected deaths		141 (34.0%)		100 (21.1%)		224 (28.5%)		465 (27.7%)
Linear trend for DPP	*p* = 0.182							
Deaths due to traffic accidents in total								
SMR (95% CI)	12.73 (12.10–13.37)	10.07 (9.50–10.65)	12.02 (11.38–12.65)	9.67 (9.09–10.24)	10.71 (10.19–11.24)	8.25 (7.77–8.73)	11.73 (11.38–12.07)	9.25 (8.94–9.55)
Observed number of deaths	1538	1217	1385	1105	1595	1203	4518	3525
Expected number of deaths *		1538		1373		1571		4482
Number (percentage) of DPP relative to expected deaths		321 (20.8%)		268 (19.5%)		368 (23.4%)		957 (21.4%)
Linear trend for DPP	*p* = 0.080							

Abbreviations: SMR—standardized mortality rate per 100,000 population; CI—confidence interval; DPP—deaths prevented or postponed. * Assuming that death rates by age groups from 2010 persist in 2015.

**Table 3:** Incidence rate ratio (IRR) for deaths due to transport accidents by health care resources (HCR), gender, age and temporal trend for out-of-hospital deaths, fatal hospitalized cases and for total deaths from accidents.

Place of Death	Mortality Due to Transport Accidents						
	Total	Pedestrian	Pedal Cyclist	Motorcycle Rider and Three-Wheeled Motor Vehicle	Car	Heavy Transport Vehicle	Other and Unspecified Transport Accidents
Out-of-hospital deaths due to traffic accidents							
Low HCR (ref: high HCR)	1.69 (1.40, 2.05) ***	1.63 (1.40, 1.89) ***	1.23 (0.94, 1.62)	1.24 (1.05, 1.47) *	1.78 (1.35, 2.36) ***	1.25 (0.81, 1.94)	0.78 (0.50, 1.20)
Moderate HCR (ref: high HCR)	1.49 (1.21, 1.85) ***	1.39 (1.21, 1.61) ***	1.22 (0.94, 1.57)	1.15 (0.98, 1.35)	1.61 (1.17, 2.22) **	1.04 (0.77, 1.38)	1.17 (0.76, 1.81)
Men (ref: women)	3.71 (3.54, 3.89) ***	2.38 (2.20, 2.58) ***	1.65 (1.47, 1.85) ***	1.68 (1.37, 2.07) ***	2.51 (2.35, 2.69) ***	1.40 (1.24, 1.59) ***	2.36 (1.99, 2.79) ***
Age group 15–24 (ref: 0–14)	4.10 (3.56, 4.73) ***	1.68 (1.43, 1.98) ***	1.44 (1.15, 1.80) **	1.93 (1.56, 2.39) ***	3.08 (2.51, 3.76) ***	1.72 (1.19, 2.47) **	1.95 (1.57, 2.42) ***
Age group 25–44 (ref: 0–14)	3.15 (2.72, 3.65) ***	1.26 (1.07, 1.49) **	0.68 (0.59, 0.79) ***	1.08 (0.85, 1.37)	1.86 (1.49, 2.31) ***	0.79 (0.55, 1.14)	1.40 (1.09, 1.80) **
Age group 45–64 (ref: 0–14)	3.34 (2.88, 3.87) ***	1.91 (1.59, 2.29) ***	1.06 (0.90, 1.26)	0.79 (0.64, 0.97) *	1.54 (1.25, 1.89) ***	0.80 (0.57, 1.14)	1.52 (1.11, 2.07) **
Age group ≥65 (ref: 0–14)	3.48 (3.03, 3.98) ***	2.48 (2.11, 2.92) ***	2.27 (1.96, 2.62) ***	1.83 (1.43, 2.33) ***	1.84 (1.55, 2.18) ***	1.34 (0.95, 1.89)	1.75 (1.39, 2.20) ***
Linear trend for year of accident	0.95 (0.94, 0.97) ***	0.95 (0.94, 0.97) ***	1.00 (0.98, 1.02)	1.01 (0.99, 1.04)	0.94 (0.92, 0.96) ***	0.96 (0.92, 1.00)	0.95 (0.90, 1.00)
Hospitalized fatal cases of traffic accidents							
Low HCR (ref: high HCR)	0.75 (0.65, 0.87) ***	0.80 (0.70, 0.93) **	1.25 (1.08, 1.44) **	0.98 (0.82, 1.18)	0.98 (0.85, 1.13)	1.25 (0.95, 1.64)	0.59 (0.45, 0.78) ***
Moderate HCR (ref: high HCR)	0.86 (0.75, 0.99) *	0.92 (0.79, 1.07)	1.34 (1.17, 1.54) ***	1.01 (0.87, 1.19)	1.08 (0.92, 1.27)	1.33 (1.04, 1.71) *	0.81 (0.62, 1.06)
Men (ref: women)	2.46 (2.29, 2.64) ***	1.66 (1.51, 1.83) ***	1.74 (1.55, 1.96) ***	1.44 (1.23, 1.70) ***	1.72 (1.54, 1.93) ***	1.27 (1.13, 1.43) ***	2.44 (2.13, 2.80) ***
Age group 15–24 (ref: 0–14)	2.13 (1.94, 2.34) ***	1.29 (1.12, 1.47) ***	1.13 (0.93, 1.36)	1.59 (1.13, 2.22) **	1.66 (1.44, 1.91) ***	0.96 (0.72, 1.29)	1.64 (1.34, 2.00) ***
Age group 25–44 (ref: 0–14)	1.24 (1.11, 1.39) ***	0.73 (0.65, 0.83) ***	0.43 (0.38, 0.49) ***	0.78 (0.56, 1.09)	0.82 (0.70, 0.94) **	0.47 (0.38, 0.58) ***	0.95 (0.77, 1.18)
Age group 45–64 (ref: 0–14)	1.75 (1.56, 1.97) ***	1.11 (0.89, 1.40)	0.69 (0.59, 0.81) ***	0.68 (0.54, 0.85) ***	0.94 (0.82, 1.08)	0.57 (0.44, 0.73) ***	1.45 (1.19, 1.78) **
Age group ≥65 (ref: 0–14)	3.46 (3.05, 3.92) ***	2.74 (2.17, 3.46) ***	1.64 (1.37, 1.97) ***	1.49 (1.23, 1.80) ***	1.71 (1.49, 1.96) ***	1.17 (0.85, 1.62)	1.81 (1.41, 2.32) ***
Linear trend for year of accident	0.95 (0.93, 0.96) ***	0.96 (0.94, 0.98) ***	1.00 (0.97, 1.03)	0.97 (0.94, 0.99) *	0.95 (0.93, 0.97) ***	0.96 (0.93, 0.98) ***	0.97 (0.92, 1.02)
Deaths due to traffic accidents in total							
Low HCR (ref: high HCR)	1.25 (1.09, 1,43) **	1.19 (1.03, 1.38) *	1.41 (1.13, 1.76) **	1.10 (0.98, 1.24)	1.58 (1.28, 1.97) ***	1.32 (0.85, 2.04)	0.68 (0.45, 1.02)
Moderate HCR (ref: high HCR)	1.21 (1.03, 1.42) *	1.14 (0.99, 1.32)	1.40 (1.12, 1.74) **	1.08 (0.96, 1.22)	1.47 (1.11, 1.93) **	1.14 (0.82, 1.58) ***	0.99 (0.63, 1.53)
Men (ref: women)	3.64 (3.49, 3.80) ***	2.36 (2.18, 2.54) ***	1.86 (1.65, 2.09) ***	1.99 (1.58, 2.51) ***	2.60 (2.45, 2.77) ***	1.41 (1.27, 1.55) ***	2.94 (2.58, 3.36) ***
Age group 15–24 (ref: 0–14)	4.37 (3.88, 4.92) ***	1.68 (1.48, 1.89) ***	1.23 (1.02, 1.48) *	2.26 (1.86, 2.76) ***	3.38 (2.86, 4.00) ***	1.57 (1.17, 2.11) **	2.16 (1.73, 2.70) ***
Age group 25–44 (ref: 0–14)	3.22 (2.83, 3.68) ***	1.33 (1.16, 1.51) ***	0.58 (0.49, 0.69) ***	1.28 (1.03, 1.60) *	2.04 (1.67, 2.50) ***	0.76 (0.56, 1.03)	1.55 (1.21, 1.98) ***
Age group 45–64 (ref: 0–14)	3.79 (3.33, 4,32) ***	2.18 (1.89, 2.50) ***	1.01 (0.82, 1.24)	0.79 (0.68, 0.91) **	1.79 (1.47, 2.17) ***	0.81 (0.62, 1.05)	1.99 (1.53, 2.59) ***
Age group ≥65 (ref: 0–14)	5.16 (4.60, 5.80) ***	3.67 (3.21, 4.20) ***	2.24 (1.85, 2.71) ***	1.72 (1.51, 1.95) ***	2.30 (1.92, 2.74) ***	1.34 (1.01, 1.77) *	2.14 (1.66, 2.77) ***
Linear trend for year of accident	0.95 (0.93, 0.96) ***	0.95 (0.93, 0.96) ***	1.01 (0.99, 1.04)	0.97 (0.95, 0.99) *	0.93 (0.91, 0.95) ***	0.93 (0.89, 0.97) ***	0.95 (0.90, 1.00)

Separate models for each transport accidents group by place of death including the effects of health care resources, gender, age, time trend. Abbreviations: HCR—health care resources; ref—reference. *** *p* ≤ 0.001; ** *p* ≤ 0.01; * *p* ≤ 0.05.

**Table 4:** Incidence rate ratio (IRR) for deaths due to transport accidents by health care resources (HCR) (reference category: moderate HCR) and for out-of-hospital deaths (reference hospital deaths) and by type of accident.

Place of Death	Mortality Due to Transport Accidents						
	Total	Pedestrian	Pedal Cyclist	Motorcycle Rider and Three-Wheeled Motor Vehicle	Car	Heavy Transport Vehicle	Other and Unspecified Transport Accidents
Low HCR (ref: high HCR)	1.25 (1.11, 1.42) ***	1.26 (1.13, 1.41) ***	1.24 (1.03, 1.50) *	1.16 (1.00, 1.33) *	1.45 (1.20, 1.76) ***	1.27 (0.87, 1.84)	0.68 (0.50, 0.94) *
Moderate HCR (ref: high HCR)	1.19 (1.03, 1.38) *	1.16 (1.04, 1.31) **	1.28 (1.08, 1.52) **	1.09 (0.95, 1.25)	1.38 (1.09, 1.75) **	1.11 (0.86, 1.43)	0.98 (0.70, 1.36)
Out-hospital deaths (ref: hospitalized fatal cases)	1.68 (1.45, 1.93) ***	1.32 (1.16, 1.51) ***	1.10 (1.03, 1.18) **	1.20 (1.11, 1.30) ***	1.74 (1.54, 1.97) ***	1.50 (1.32, 1.70) ***	1.18 (1.01, 1.38) *

Separate models for each transport accidents group including the effects of health care resources and place of death adjusted for gender, age, time trend. Abbreviations: HCR—health care resources; ref—reference. *** *p* ≤ 0.001; ** *p* ≤ 0.01; * *p* ≤ 0.05.

## Data Availability

Data were collected from publicly archived datasets analyzed or generated during the study and presented in Table 1.
